# Experiences of mobility for people living with rheumatoid arthritis who are receiving biologic drug therapy: implications for podiatry services

**DOI:** 10.1186/s13047-017-0195-4

**Published:** 2017-03-16

**Authors:** Lucy Sanders, Margaret Donovan-Hall, Alan Borthwick, Catherine J. Bowen

**Affiliations:** 1 Faculty of Health Sciences, University of Southampton, Highfield Campus Building 45, University Road, Southampton, Hampshire SO17 1BJ UK; 2 NIHR Musculoskeletal Biomedical Research Unit, University of Oxford, Oxford, UK

**Keywords:** Rheumatoid arthritis, Biologic therapy, Mobility, Footcare

## Abstract

**Background:**

Despite significant advancements in new treatment modalities for rheumatoid arthritis with biological therapies, foot complications remain a disabling and common feature of the disease**.** In this study the aim was to explore and describe the personal experiences of people with rheumatoid arthritis in receipt of biologic treatments in a bid to understand the impact of this form of medication on their mobility.

**Methods:**

An interpretative phenomenological analysis (IPA) was undertaken to explore in depth the individual experience of rheumatoid disease through personal accounts of the patient journey spanning both ‘before’ and ‘after’ the instigation of biologic therapy. A purposive sampling strategy was adopted and in-depth semi structured interviews used to facilitate rich, detailed interview data exploring the lived experiences of individuals undertaking biological therapy and the changes to mobility experienced as a result. Thematic analysis was employed with an IPA framework to identify key meanings, and report patterns within the data.

**Results:**

Five people with rheumatoid arthritis participated in the study. The mean disease duration was 20.2 years (range: 6 -32) and all were being treated with biologic therapies. Four key themes emerged from the data: 1) Life before biologic treatment, depicted in accounts as a negative experience characterised by painful and disabling symptoms and feelings of hopelessness. 2) Life with biologic treatment, often experienced as a life changing transition, restoring function and mobility and offering renewed hope. 3) Sense of self, in which the impact of rheumatoid disease and the subsequent changes arising from biologic therapy reveal a profound impact on feelings of personal identity both pre and post biologic therapy; an effect of footwear on self-image emerges as a dominant sub theme; 4) Unmet footcare needs were evident in the patient narrative, where the unrelenting if diminished impact of foot pain on mobility was viewed in the context of problematic access to foot health services.

**Conclusion:**

Whilst the findings from this study mirror those within the existing literature, which report improvements in physical function related to biological therapy, foot problems clearly remained an unremitting feature of life for patients with rheumatoid disease, even when in receipt of biologics.

**Electronic supplementary material:**

The online version of this article (doi:10.1186/s13047-017-0195-4) contains supplementary material, which is available to authorized users.

## Background

Rheumatoid arthritis (RA) is the most common form of chronic inflammatory polyarthritis [1] with a relatively constant prevalence across many developed populations worldwide, including the UK at 0.5-1.0% [2]. Improved understanding of the molecular pathways responsible for driving the disease are demonstrated in the variety of biologic treatments now available, each with varying immunological targets and modes of action [3]. There is a growing body of evidence demonstrating improvements in patient outcome measures in those taking biologics [4–7], although much of this evidence is derived from quantitative research studies, with relatively little attention focused on the individual patient experience.

The prevalence of foot involvement and foot pain in RA is well documented in the literature with an estimated range of 35-70% [8, 9]. Despite the significant advances in new treatment modalities for RA, such as the availability of disease modifying anti-rheumatic drugs (DMARDs) and biologic treatments, foot complications remain a disabling and common feature of life for many patients [9]. Indeed, the presence of forefoot pain (63.9%) and ankle pain (42.7%) was highlighted by Otter et al. [9] from a cross sectional survey of patients with RA (*N* = 585). However, these estimates may actually be higher, as the prevalence of foot involvement and the extent of foot symptoms in the RA patient group can be under-estimated [9, 10].

Foot complaints and impaired foot function occurring in both the early and chronic stages of RA have been shown to severely limit an individual’s daily activities, in particular reducing ambulation and functional mobility [11–14]. Pain from rheumatoid forefoot disease has also been linked to disability in a number of weight bearing activities [14]. Indeed, in a survey by Grondal et al. [12] forefoot symptoms were reported as a primary cause in walking impairment in the rheumatoid population, significantly more so than symptoms from either the knee or hip joints (*N* = 1000). Consequently, investigators have begun to highlight the negative impact of RA related foot disease on quality of life (QOL) [15, 16] although few studies have demonstrated this unequivocally [17].

A new paradigm for the management of foot manifestations of RA has been proposed, which includes tighter disease control through the use of biologic treatments [18]. Other investigators have shown that patient reported foot pain and disability may decrease in people who have RA (PwRA) following 12 weeks of anti-TNFα treatments compared to those on conventional DMARDs [19]. Conversely, in a study comparing self-reported foot pain in PwRA treated with and without anti-TNFα, Otter et al. [9] found that foot pain was significantly increased within the anti-TNFα treated group (*P* = 0.012). The authors suggest that the success of the TNF inhibitors may encourage a greater degree of mobility in the patients receiving it, which in turn may *increase* foot symptoms [7, 9, 20]. This may be evidenced by observed improvements in gait amongst PwRA successfully treated with anti-TNFα [21].

From an extensive review of the literature it is clear that very little evidence is available to confirm these theories. Three key qualitative publications have been identified within the area of biologics and experiences of PwRA. Marshall et al. [22] reported improvements in physical function, pain and well-being using interview data (*N* = 19). They indicated a greater range of benefits from biologic treatments than those routinely measured in quantitative research [22]. Arkell et al. [23] found that participants were very positive about anti-TNFα medication, which they felt led to dramatic changes in their physical symptoms. Equally, Linden and Bjorklund [20] concluded that successful treatment with anti-TNFα medication led most participants to experience dramatic improvements in the quality of their lives, resulting in a return to leisure interests, enhanced social activity and improved or continued productivity in education and/or occupation. However Linden and Bjorklund also cautioned that such dramatic changes may risk overuse and consequent strain [20].

A constant challenge for service providers remains the appropriate targeting of resources, which ideally would be guided by patient reported experiences, particularly where pharmacological management is especially effective at reducing pain and disability. This is very notable in relation to patient experiences of mobility in those who have commenced anti -TNFα drug therapy. As a result, the aim of this study was to gain a deeper understanding of the experiences of PwRA who are receiving biological therapy treatment in relation to their lower limb mobility.

## Methods

A qualitative research study design was employed to enable a deep exploration of the lived experiences of individuals with RA in receipt of pharmacological treatment with biologic therapies, which would examine the impact of these treatments on their world. Interpretative phenomenological analysis (IPA) provided the most appropriate approach. IPA is a qualitative approach that involve an idiographic focus which is concerned with providing insights into how a individual in a specific context make sense of a particular issue (phenomenon) [24]. In this study, this involved trying to understand the unique personal meanings and actual experiences of PwRA patients attempting to regain and maintain mobility whilst receiving biological therapy treatment. This exploratory idiographic approach was adopted using semi structured interviews, and it deployed a chronological narrative mapping of experiences before the onset of RA, after its onset, and the journey through the transition to biologic therapies and their impact, thus spanning a significant timeframe in the participants’ lives.

### Ethical approval

Full ethical approval for the study was granted by the Faculty of Health Sciences, University of Southampton Ethics Committee via the online approval system ERGO (Ethics and Research Governance Online). Our original proposal had a tighter inclusion criteria for specific anti-TNF therapies, however this substantially reduced our potential recruitment sample and therefore we widened our criteria to include PwRA who were receiving any biologic therapy. The amendment to extend the sample to include patients receiving all current biologics rather than just one specific branch, anti-TNFα was approved.

### Participants

Participants were recruited through branch membership of the National Rheumatoid Arthritis Society (NRAS) using the Salisbury and North Hampshire branches. All members of NRAS have rheumatoid arthritis. Potential participants were approached with a short presentation that explained the study by the primary investigator (LS) at the meeting of each branch. Those interested in joining the study were provided with an information sheet along with the contact details of the primary investigator (LS). Individuals volunteering to join the study then contacted the primary investigator (LS) for additional information, to have any further questions answered, and to be pre-screened against the inclusion criteria.

A purposive sampling strategy was undertaken, consistent with the qualitative paradigm and study design adopted. This enabled the study to capture insightful and meaningful data exploring individual patient experiences, and to note any common experiences across the sample. Participants were included if they had a confirmed diagnosis of rheumatoid arthritis; were currently receiving biologic treatment, where the duration of biologic treatment was over 6 months; where they were aged 18 or over and were able to give consent to participate in the study. Participants were excluded if they had a diagnosis of inflammatory arthritis other than rheumatoid arthritis, or were unable to speak or understand English.

### Procedure

Individual, face-to- face, semi-structured interviews were undertaken, generating data which was transcribed verbatim prior to analysis. Each interview was conducted by one researcher (LS) to enable consistency in approach, whilst also capturing individual accounts and narratives. An interview topic guide (Additional file 1) was used to enable a degree of continuity across the interviews, whilst acknowledging the need to capture the individual experiences of each participant. To begin the interview a broad open question about the participant’s experiences of everyday life with rheumatoid arthritis prior to biologic therapy was used. The answers were then followed up with exploratory questions, probes and prompts. As there has been little published work on patient experiences of RA when undergoing biologic therapy, the topic guide was informed by, and constructed from, the clinical experience of the research team. Participants were able to choose the venue of the interviews, either in their own homes or in a room at the University of Southampton. The interviews lasted between 40-60 min each and all were audio-recorded. Interview audio recordings were transcribed verbatim. One researcher (LS) read and re-read transcripts several times to enable familiarity and immersion of the data. To verify the accuracy of the researcher’s transcription, the first participant was offered a copy of their transcribed interview to check for veracity, accuracy and to ensure rigour which was confirmed by the individual participant (respondent validation).

Thematic analysis was used to analyse the data within an IPA framework focussing on the individual lived experience. One researcher (LS) undertook a line by line analysis of the experiences and understanding of each participant. These initial notes were used to develop codes which informed the further development of themes and sub-themes. Codes were generated from transcripts by noting recurring words or comments of interest. Following this process all codes were then laid out and collated into groups of similar features which served as potential themes. The emerging themes were discussed and viewed by the wider research team (LS, MDH, CB) to verify the themes, identity any additional areas of interest and agree the themes. This involved discussion and looking at patterns across the data, but did not involve each member analysing the data individual and then comparing the finding. To further develop the themes and make clear links to the transcripts, extracts from the transcripts were collated into a table for ongoing analysis (Additional file 2). Potential themes were then discussed again with the research team (LS, MDH, CB) to identify any alternative interpretations. The process of verifying themes as a team provided a more rigorous approach, different perspective and agree on the final themes.

## Results

The study recruited seven participants, two male and five female from the two NRAS branches. However one female participant was unable to attend interview and therefore withdrew. One further interview was initially postponed and the participant opted to withdraw. Five interviews were finally conducted that included one male and four female participants (mean age 64, range 35 to 78 years). The average duration of disease was 20.2 years (range 6–32 years). The average duration of receiving biologic therapy was 5.4 years (range 2005–2013), although several participants had been on other biologics before and these were brought up in the interviews. At the time of the interviews, the participants were experiencing varying degrees of effectiveness of their biologic treatments. Two participants were having infusions of Rituximab, one of which was waiting to change to Abatacept due to poor efficacy. Two participants were having Humira injections and one patient was having Enbrel injections. Three participants had no prior history of biologic drug therapy, whilst one participant was awaiting the start of a third different biologic and another was starting on a fourth.

The analysis identified four key themes: 1) Life before biologic treatment, 2) life with biologic treatment, 3) sense of self, and 4) podiatric implications. Each theme is discussed and supported with direct extracts drawn from the transcripts. Participants are assigned numerical identifiers to ensure confidentiality.

### Life before biologic therapy: “If you did too much you went to bed”

This theme related to individuals experiences and key events in the years prior to the receipt of biologic therapy, including the physical, social and emotional impact. Some of these accounts convey the experience of a dark period in participants’ lives, associated with “survival” and a time “not to visit again”.

Each account captured the contrast between life prior to biologic therapy and that after it, providing a context within which to comprehend the extent of the impact of the drugs. Symptoms of pain were common to all the participants, albeit to a varying degree. However, the intensity of the impact of living with pain is captured in the concise but descriptive and powerful phrases employed by some of the female participants, all of whom were diagnosed more than 10 years previously.

*“Every single joint is on fire” (P01)*

*“Absolute agony; if you just touched me I would scream with the pain” (P02)*

*“A normal day was agony” (P03)*

*“The damage comes with really severe pain” (P05)*

All participants clearly experienced the undesirable and troublesome impact of physical symptoms arising from rheumatoid arthritis, with each identifying a range of distressing and disabling restrictions, including ‘tiredness’ and ‘exhaustion’. One short data extract particularly captures the extent of the disabling impact of their symptoms.

*“[I] usually went back to bed after breakfast because I needed to” (P03)*

Joint and limb stiffness as much as pain were viewed as disabling, preventing the accomplishment of the simplest of everyday tasks.

*“When I got up in the mornings it took me about 20 min to be able to walk normally…my arms stopped working; I actually couldn’t raise them over shoulder height” (P04)*

Participants also described the experience of becoming less active, or diminishing physical capabilities, leaving a sense of loss and requiring a re-evaluation of self-image and identity. For example, those participants who had previously participated in sport had been forced to abandon it altogether.

*“I was playing tennis at the time, just got back into tennis, so I stopped doing that, um, and I used to run, just road running - I couldn’t do any of that. And then just general things, getting out on the bikes or going swimming, that all stopped.” (P04)*

Strong emotional feelings were expressed when discussing life prior to being treated with biologic therapy. One participant in particular (P05) found it very ‘emotional and difficult’ talking about the time around her diagnosis, becoming quite tearful on several occasions and expressing suicidal thoughts.

*“It had got so bad [pauses] I could understand how somebody could top themselves…..you know, the pain was just so bad” (P01)*

*“You just want to lay in bed and [pause] just be left alone and [pause] die in your own way, if you know what I mean, ‘cos the pain is so excruciating” (P02)*

Loss of sense of self and self- identity were very evident, where comparisons were drawn between the person they had been (active, sporty) to that which they had become (disabled).

*“I’d gone from being very active to sitting on the stairs crying” (P05)*

### Life with biologic therapy: “I’m doing more than I thought possible”

This theme relates to participant narratives reporting experiences since commencing biologic therapy. All participants reported the remarkably liberating effects of what was described as a ‘miracle’ treatment, even referring to life as ‘glorious’ in some cases.

*“It has just kind of given me back my life…..and that is such a good thing.” (P01)*

Each narrative was marked by the use of highly evocative and descriptive language suggestive of a dramatic life-changing event, evident when the biologic treatment was effective.

*“When I had this Enbrel, it was just like being reborn actually, quite life changing” (P02)*

*“God, it was incredible, absolutely incredible….it was just the most amazing thing…I think that’s what is amazing about it, is that you appreciate things more…it’s like a gift” (P05)*

The startling effectiveness of the biologic therapy also enabled participants to re-engage with physical activities that were presumed lost for ever, prompting them to take every opportunity to do so.

*“I can go and do things, so any chance now I get, I go!” (P01)*

*“I can even push a lawn mower now!” (P02)*

*“I ran the great south run this year” (P04)*

*“I walk up the hill, it’s in a valley, and it’s a really steep hill and I’ve been on health walks with the local health group walk. And I walk on my own, and I walk for an hour (P05)*

Unsurprisingly, the ability to resume physical activities that had seemed unlikely prior to biologic therapy brought a sea change in mood and an uplift in psychological well-being.

*“Well I think it, umm, I think it cheers you up a lot. A lot of it is in the mind, and when you are happy your pain is less anyway, and you can do more….So mentally it makes your life much better” (P02)*

*“I walked miles, and it was just incredible and I felt so much better in myself” (P05)*

Not all the respondents experienced such dramatic improvements, particularly those who had tried several biologics previously, but had experienced a reduced tolerance of the drug or a failure in efficacy, or, in some cases, a regression and resumption in symptoms, with an understandable loss of optimism.

“*What was so difficult was that 9 months later the drug (Infliximab) almost overnight stopped working…It was almost worse than having it, it was like re-visiting where I was in the first place…Part of me always thinks wouldn’t it be wonderful if I can find something like Remicade (Infliximab) again, that was 100%, that put me into remission that I know is possible” (P05)*

*“Enbrel must have helped with that (walking) but the Rituximab hasn’t quite managed to keep it up. I’ve gone back a bit, quite a bit really…Feet, my feet seem to have got much worse….whether I can blame the Rituximab for that I don’t know, but it certainly has got worse” (P03)*

Equally, in some instances respondents had experienced a remission in symptoms of pain and stiffness, and had avoided irreversible joint damage and deformities, but nevertheless had been left with some residual effects and limitations to everyday activity, often met with resignation.

*““I think every joint has gone back to roughly being ok*…*I think the biggest thing for me is the fatigue…There are times when I can’t keep playing with them (children) and I do have to come and sit down, which gets me a bit frustrated. So, so [pause] yeh it has become the ‘norm’ so I don’t think about it as much and I have just accepted that I have to come and do it” (P04)*

### Sense of self: “You’re a woman & want to feel nice”

Bury’s classic work (1982) on biographical disruption powerfully captured the impact of the onset and deterioration of rheumatoid disease on personal identity and self-image, and Williams’s subsequent work on biographical reconstruction (1984) took account of the normalisation process following successful medication, although neither works were able to address the changes heralded by the modern use of biologic therapy [25, 26]. Unsurprisingly, notions of self-identity and self-image were, as evidenced in the data presented here, transformed with the advent of biologic therapy, although established signs of disease continued to result in feelings of being labelled.

With deformity established as a residual effect of earlier active disease, participants reported a self-consciousness generated by the knowledge that visible signs of their disease, such as found in the hands, were highly noticeable to others around them.

*“I was standing in the supermarket queue and I saw that someone else had the same hands as me and I knew that she’d got rheumatoid too and it’s like I’m an individual, I don’t wanna be part of this club, I don’t want this label” (P05)*

Individual sense of self-image may be drawn from, and constructed around, a sense of how individuals are seen by others, which was evident in the data and seemed to reflect a desire to be viewed as ‘normal’ in one’s public self (‘normalisation’ as a form of coping, bracketing off the impact of the disease – see Williams [27]), which also maps with William’s reflections [27] on ‘corporeal dysappearance (dysfunctional appearance)’.

*“I do try and walk without a stick around the town, so that I’ve not always got a stick. And sometimes you think, ooh, why didn’t I get the stick out the car? But the point is, umm, that way [pause] I’m just normal! [laughs]” (P01)*

Self-image, personal identity and the participants’ ‘public self’ was most evidently affected in accounts concerning the individuals’ social life. For example, one participant spoke about friends who had met partners via online dating sites, contrasting this with her own situation in which she felt unable to consider any such activity as realistic or feasible, given her physical appearance.

*“It can be embarrassing, [pause] I mean, [pause] I’d never do this online dating, cos I’d have someone have to see me first [sic]. See what my hands are like. I couldn’t have someone never see me and then see things like disabled crippled hands” (P02)*

Another element of concern expressed about the effects of changed physical appearance was the focus on the accompanying special footwear often prescribed for patients with particular foot problems, such as deformity. In such cases, it was no longer possible to walk comfortably, or fit into, ‘ordinary’ footwear (that which might be purchased in a high street shoe shop). However, prescription orthopaedic footwear is notable for its bulky, functional appearance, with very little aesthetic consideration in its design, a matter of particular concern to female patients. Wearing such footwear therefore marks out the individual, highlighting the disability.

*“How can you wear things like that, even no matter how much pain I was in there was no way I’m gonna be seen dead in them, I’m not even gonna be put in my coffin in one of those on me” [sic] (P02)*

*“I have to wear these (pause) ghastly things (laughs) to get any degree of comfort” (P03)*

*“Have you ever seen those great big shoes? I said I can’t wear that, that is insulting really. I still wanna be, I’m still a woman!” (P02)*

### Theme 4. Podiatric Implications: “They never tell you anything about your feet”

In some instances the reported beneficial effects of biologic therapy did not extend to foot pain. Whilst global pain had diminished, foot pain remained. This may have been due to established deformity acquired prior to biologic therapy, or a consequence of foot surgery.

*“The pain has improved all over with the Enbrel injections, yeh,that I can’t deny. But my pain in my feet, my feet are still bad because as I say I’ve had all the operations, and the deformity.” (P02)*

Access to allied healthcare professionals was an area of importance to all participants; all the participants reported finding it difficult to access services, due to a lack of referral, with the common consensus being that “you’ve got to go and ask for it” (P01). In addition, it is clear that a lack of inter-professional co-ordination hampers effective and timely referral, and the criteria adopted for referral may appear iniquitous.

*“Umm to be honest I don’t know if it’s something they do with everyone [refer to PT & OT] or just, they did say my RA was quite severe and had come on suddenly. I don’t know if that’s why they did it or because I was asking them for help. I think at the time I was asking for things that I could do to try and keep myself more mobile.”(P04)*

Access to podiatry services specifically appears particularly problematic, possibly due to a lack of awareness by other professionals of the roles within podiatry.

*“…I have never seen a podiatrist … I’ve never been recommended to see one…” (P01)*

*“They [nurses and physicians] feel your knees and sorts of things and see how you can move your ankles but they never seem to bother much about the feet” (P02)*

*“I’m sure I mentioned it to my GP and rheumatologist umm but I think that was just part of the overall symptoms and my feet individually weren’t really looked at” (P04)*

## Discussion

Exploring the lived experience of individuals with rheumatoid arthritis in receipt of biologic therapy marks a step forward from the work of Bury [25] and Williams [26], in that it allows a glimpse into the world of patients whose lives have been transformed by these new drugs. Whereas Bury and Williams established and developed the concepts of biographical disruption and biographical reconstruction, the advent of biologics treatment has opened the door to a new dimension in the subsequent normalisation process. In this paper the focus has been on those elements of individual life experience which impact on mobility and self-image, capturing both the life before and the life after biologic therapy. In doing so it allows the links between transformative drug therapy use and personal self-identity to be explored, and to examine ways in which this modifies or reflects the processes outlined by Bury and Williams in their seminal works [25, 26]. The participant narratives in this study resonate strongly with those identified by Bury, Williams and others in examining the impact of chronic illness and the disabling effect on mobility and physical changes that effect self-image. Following biologic therapy, the narratives assume a quite different course – an accelerated form of normalisation which brings its own unique problems; notably overuse of joints, ligaments and soft tissues in previously damaged, deformed and vulnerable feet.

Broadly, the experiences of biologic therapy reported by the participants suggested a dramatic and significant improvement in symptoms, enabling a return to near normal activity in some instances. These findings are largely consistent with Marshall et al. and Arkell et al. [22, 23], although they explored the effects of anti- TNFα only, rather than the wider group of biologic drugs. Unsurprisingly, the changes noted in those starting biologics were linked to perceived improvements in quality of life (QOL). Similar results were also found in a range of recent quantitative studies, with anti- TNFα treatment shown to significantly improve health related QOL [4, 28].

Another important finding from our study was the dramatic improvement in the degree of physical activity and participation possible as a result of biologic therapy. This is consistent with the conclusions reached by other investigators [4, 9, 20, 28].

It is clear that the value of the physical improvements resulting from biologic therapy was transformative, bringing with them a feeling of well-being through re-engaging in physical activity. Linden and Bjorklund [20] also reported improved physical and mental well-being in patients treated with anti- TNFα, as did Loeppenthin et al. [29], where patients were treated with biologics.

In the accounts presented here, it is clear that those participants who experienced a return to near normal physical activity following biologic therapy appeared to normalise rapidly, to the extent that their expectations of life were raised to that of life as it was before the advent of disease. To feel restored to normal function and ability naturally encouraged the participants to attempt to engage in activities in the way they may have done prior to the onset of RA. For those patients with advanced or chronic long standing disease, this may not be a realistic expectation, given the extent of muscle atrophy and fatigue or deformity that may have accumulated over a number of years prior to commencing biologics.

PwRA overstraining themselves due to receding symptoms and a resultant normalisation is highlighted by Linden and Bjorklund [20]. Although patients may need to learn to balance their ‘re-born’ lifestyle with enough rest, our findings agree with those of Linden and Bjorklund [20] and suggest that further input from allied health professionals may be beneficial in assuming a more measured approach to regaining activity. For PwRA who are positive responders to biologics, those with early access and recent diagnosis may require different interventions to those with long-standing chronic disease, as the latter may have a greater level of tissue damage, deformity or history of surgery. Those PwRA with chronic disease who ‘over-do-it’ may experience such complications as tissue viability loss due to increased activity involving vulnerable structures, as described by some participants.

In such circumstances podiatrists are able to monitor areas at risk of ulceration occurring from increased demand. Avoiding ulceration is critically important, given the enhanced risk of infection associated with taking biologic drugs, and the potential for flares if treatment is temporarily stopped. Prescription foot orthoses may also provide additional protection and help to minimise the dangers of overuse of the feet.

A lack of choice surrounding footwear could impact upon the negativity expressed regarding the aesthetics of the orthopaedic footwear issued to some patients. This is already acknowledged in the current literature by Williams et al. who suggest that footwear may reinforce a negative self-image [30]. Similar themes around identity and footwear were also found by Farndon et al, however they also highlighted conflict in what podiatrists and the people that they support and treat look for in a shoe [31]. Biologics might make a person feel better physically, but established deformity is irreversible and a damaged self-image may therefore persist. Footwear aesthetics may need to be acknowledged and addressed by clinicians if patients are to effectively use prescription shoes.

Importantly, if we are to acknowledge that effective response to biologics can lead to improved physical function and increased physical activity, combined with improved foot function, and that ‘over use’ may be an unintended consequence, then a role in closely monitoring foot health in these patients is clear.

It is also clear that podiatrists may provide that service, as is acknowledged in both the literature and in clinical guidelines, which recommend access to a specialist in foot health such as a podiatrist for assessment and review of foot health needs [16, 32, 33]. Despite this, there appears to be a lack of access to podiatry services for PwRA and this has been consistently reported within the recent literature relative to the UK [34, 35] and other countries [13, 36]. Whilst new models of foot care for PwRA have been proposed [37, 38] further exploration of these is recommended for implementation of such strategies within routine practice.

### Study limitations

A reflection on the study methodology is relevant, given the sample size was less than expected. By incorporating the experiences of a wider group of people more themes may have been generated, or the current themes identified may have been seen as more or less significant. It is likely that age at diagnosis, disease duration and experiences of illness before accessing biologics are key variables. Although this varied in participants for this study, a larger sample would have enabled comparisons between variables such as differing lengths of disease duration. Finally, recruitment from a support group for PwRA could mean that the participants were well motivated and may have influenced the data obtained. If we had sought participants outside of this group we may have resulted in a different narrative.

## Conclusion

The findings from this study advance the work of Bury and Williams, and highlight the personal and social impact of biologic therapy on patient mobility. Most participants experienced dramatic changes in their daily lives due to a reduction in symptoms from effective biologic treatment. However, it is clear that there are consequences to this improvement in function, in that over exertion may lead to further foot problems, highlighting a need for specialist foot health care monitoring and improvements in patient education which could be provided by podiatrists.

## Additional files

Additional file 1: Topic guide for semi-structured interviews. (DOCX 13 kb)
Additional file 2: Themes and sub-themes. (DOCX 13 kb)

## Abbreviations

NICE: National Institute for Health and Care Excellence
NRAS: National Rheumatoid Arthritis Society
PwRA: People living with rheumatoid Arthritis
RA: Rheumatoid arthritis
