# Supplementary material for: Experiences of mobility for people living with rheumatoid arthritis who are receiving biologic drug therapy: implications for podiatry services
Source: J Foot Ankle Res. 2017 Mar 16;10:14. doi: 10.1186/s13047-017-0195-4 (PMC5356260; doi:10.1186/s13047-017-0195-4)
Supplement: Additional file 1: — Topic guide for semi-structured interviews. (DOCX 13 kb) [file 13047_2017_195_MOESM1_ESM.docx]

# Additional file 1

TOPIC GUIDE FOR SEMI-STRUCTURED INTERVIEWS

Topic prompts:

1. Can you tell me about your experiences of everyday life when you were first diagnosed with rheumatoid arthritis?
2. Please can you tell me about what a normal day was like for you prior to starting on your biologic drug therapy?
3. Can you describe to me your experiences related to your mobility levels before you started on your biologic drug therapy?
   1. Around the house
   2. Going outside
   3. Activities
   4. Weekly tasks ie supermarket
4. What are your experiences of health and how you feel since you have been taking biologic drug therapy?
   1. Positive
   2. Negative
5. Do you remember noticing any changes to your activity and mobility levels are starting your biologic drug therapy?
6. Please talk me through any discussions you may have had with other health care professionals?
