# Supplementary material for: Experiences of mobility for people living with rheumatoid arthritis who are receiving biologic drug therapy: implications for podiatry services
Source: J Foot Ankle Res. 2017 Mar 16;10:14. doi: 10.1186/s13047-017-0195-4 (PMC5356260; doi:10.1186/s13047-017-0195-4)
Supplement: Additional file 2: — Themes and sub-themes. (DOCX 13 kb) [file 13047_2017_195_MOESM2_ESM.docx]

# Additional file 2

THEMES AND SUB- THEMES

1. “If you did too much you went to bed” - LIFE BEFORE BIOLGICS

1.1 Painful symptoms

1.2 Physical restrictions

1.3 Stopping activities

1.4 Social restrictions

1.5 Emotional effects

2. “I’m doing more than I thought possible 3-4 years ago!” - LIFE WITH BIOLOGICS

2.1 Dramatic Change

2.2 Appreciation

2.3 Psychological wellbeing – Return to activities

2.4 Multiple biologics

2.5 Physical improvements

2.6 Return to activities

3. “Whatever age you are, you’re a woman & want to feel **nice**” - SENSE OF SELF

3.1 Self image

3.2 Invisibility

3.3 Embarrassment

3.4 Footwear (appearance)

4.”They never tell you anything about your feet” - PODIATRIC IMPLICATION 5.1 Ongoing foot involvement

5.2 Foot surgery

5.3 Access to services

5.4 Podiatric awareness
